# The effect of imipenem and diffusible signaling factors on the secretion of outer membrane vesicles and associated Ax21 proteins in *Stenotrophomonas maltophilia*

**DOI:** 10.3389/fmicb.2015.00298

**Published:** 2015-04-14

**Authors:** Simon Devos, Laurence Van Oudenhove, Stephan Stremersch, Wouter Van Putte, Riet De Rycke, Gonzalez Van Driessche, Jolien Vitse, Koen Raemdonck, Bart Devreese

**Affiliations:** ^1^Laboratory for Protein Biochemistry and Biomolecular Engineering, Department of Biochemistry and Microbiology, Ghent UniversityGhent, Belgium; ^2^Laboratory for General Biochemistry and Physical Pharmacy, Department of Pharmaceutics, Ghent UniversityGhent, Belgium; ^3^Department of Biomedical Molecular Biology, Inflammation Research Center, Ghent UniversityGhent, Belgium; ^4^Inflammation Research Center, Flemish Institute for Biotechnology (VIB)Ghent, Belgium

**Keywords:** outer membrane vesicles, *Stenotrophomonas maltophilia*, antibiotic resistance, proteomics, quorum sensing

## Abstract

Outer membrane vesicles (OMVs) are small nanoscale structures that are secreted by bacteria and that can carry nucleic acids, proteins, and small metabolites. They can mediate intracellular communication and play a role in virulence. In this study, we show that treatment with the β-lactam antibiotic imipenem leads to a dramatic increase in the secretion of outer membrane vesicles in the nosocomial pathogen *Stenotrophomonas maltophilia*. Proteomic analysis of their protein content demonstrated that the OMVs contain the chromosomal encoded L1 metallo-β-lactamase and L2 serine-β-lactamase. Moreover, the secreted OMVs contain large amounts of two Ax21 homologs, i.e., outer membrane proteins known to be involved in virulence and biofilm formation. We show that OMV secretion and the levels of Ax21 in the OMVs are dependent on the quorum sensing diffusible signal system (DSF). More specific, we demonstrate that the *S. maltophilia* DSF cis-Δ2-11-methyl-dodecenoic acid and, to a lesser extent, the *Burkholderia cenocepacia* DSF cis-Δ2-dodecenoic acid, stimulate OMV secretion. By a targeted proteomic analysis, we confirmed that DSF-induced OMVs contain large amounts of the Ax21 homologs, but not the β-lactamases. This work illustrates that both quorum sensing and disturbance of the peptidoglycan biosynthesis provoke the release of OMVs and that OMV content is context dependent.

## Introduction

*Stenotrophomonas maltophilia* is the most frequently isolated unusual non-fermenting Gram-negative bacterium in hospitalized patients (Fihman et al., 2012). It is associated with an expanding range of clinical syndromes like bacteraemia, pneumonia and soft-tissue infections. *S. maltophilia* is also one of the most common emerging multi-drug resistant organisms found in the lungs of cystic fibrosis (CF) patients, with increasing prevalence (Amin and Waters, 2014). The high mortality in immuno-compromised patients observed with these infections is associated with the intrinsic and acquired resistance of *S. maltophilia* to many of the currently used antibiotics, including those of the β-lactam carbapenem type (Fihman et al., 2012). Indeed, antibiotic stress induces the production of two different chromosomal encoded carbapenem-hydrolyzing β-lactamases, the L1 metallo- and L2 serine-β-lactamases (Van Oudenhove et al., 2012).

In some species, chromosomal encoded β-lactamases can be secreted in outer membrane vesicles (OMVs), enabling extracellular β-lactam degradation (Ciofu et al., 2000). Such OMVs are actually secreted by all Gram-negative bacteria and have different biological functions including protection of the secreted cargo, long-distance transport of toxins and virulence factors, cell-cell communication, pathogenesis, antibiotic resistance and aiding in biofilm formation (Deatherage et al., 2009; Bonnington and Kuehn, 2013; Tiwari, 2014). Recently, Ax21 was identified in *S. maltophilia* as an important OMV-associated virulence factor (McCarthy et al., 2011; Ferrer-Navarro et al., 2013). While the actual function of the Ax21 protein in *S. maltophilia* is still uncertain, studies in the closely related plant pathogen *Xanthomonas oryzae* have provided evidence for a role in motility and biofilm formation (Park et al., 2014). *Xanthomonas* Ax21, recently renamed to omp1X, is an outer membrane β-barrel protein that is secreted by the general secretion (Sec) system, and it is associated with outer membrane vesicles (OMVs) as well (Bahar et al., 2014).

In *X. oryzae* pv. *oryzicola*, Ax21 expression was found to be regulated by the diffusible signal factor (DSF) quorum sensing (QS) system (Qian et al., 2013). The DSF system is a well-established QS system involved in cell–cell signaling and the regulation of biofilm formation in *S. maltophilia* (Fouhy et al., 2007; Ryan and Dow, 2010). DSF cell-cell signaling is managed by “regulation of pathogenicity factors” (rpf) proteins, encoded in the *rpfBFCG* gene cluster (Huedo et al., 2014). The enoyl-CoA hydratase family enzyme RpfF and the long-chain fatty acyl coenzyme A ligase RpfB are responsible for the synthesis of cis-Δ2-11-methyl-dodecenoic acid, the main DSF molecule in *S. maltophilia* (Huang and Lee Wong, 2007). Extracellular DSF can be perceived by the two-component system (TCS) histidine sensor kinase RpfC, which activates the RpfG response regulator. The activated RpfG then acts as a cyclic diguanosine monophosphate (c-di-GMP) phosphodiesterase, influencing cellular c-di-GMP levels and downstream regulation events (Ryan and Dow, 2010; Tao et al., 2010). Interestingly, the *S. maltophilia* DSF molecule can also influence other species like *Pseudomonas aeruginosa* and *Burkholderia cenocepacia* (Ryan and Dow, 2010), which often co-colonize in the lungs of patients with cystic fibrosis (CF) (Twomey et al., 2012).

It is still unknown what mechanisms are responsible for OMV biogenesis, and how its content is selected. However, it is believed that it entails an active process, and based on the OMV cargo, several models have been proposed (Haurat et al., 2014). A recent study on another species within the Xanthomonadaceae, the plant pathogen *Xylella fastidiosa*, showed that OMV biogenesis (and virulence) is suppressed by the DSF system (Ionescu et al., 2014). The aim of this study was to investigate the effect of different DSFs on the secretion of OMVs in *S. maltophilia*, as well as the influence of β-lactam antibiotic treatment. We also report on the OMV-associated secretion of two Ax21 homologs and on how DSFs affect the abundance of these homologs in OMVs.

## Materials and methods

### Materials

Urea was obtained from GE Healthcare (Diegem, Belgium). Tris-HCl (UltraPure™, 1M, pH 8) was purchased from Invitrogen (Carlsbad, CA, US). Bovine serum albumin (MS grade protein standard) was purchased from Protea Biosciences Group (Morgantown, WV, US). Sequencing grade modified trypsin was obtained from Promega (Madison, WI, US). ULC-MS grade water, acetonitrile (ACN) and formic acid was procured from Biosolve (Valkenswaard, The Netherlands). Imipenem was kindly donated by Prof. M. Galleni (CIP, University of Liège, Belgium). Other chemicals and reagents were purchased from Sigma-Aldrich (St. Louis, MO, US).

### Bacterial cell culture

The imipenem-resistant *S. maltophilia* strain 44/98 (LMG 26824, a kind gift of Dr. Paola Mercuri, Ulg, Belgium) was isolated at the Clinical Microbiology Unit of the Varese University hospital in Italy. Cultures were grown aerobically overnight in Luria Broth (LB) until the stationary phase. The cell suspensions were then diluted to an OD_600nm_ of 0.2, grown until the mid-exponential growth phase (OD_600nm_ = 0.65–0.75), and then stimulated with either 25 μg/mL imipenem, 1 mM *Stenotrophomonas maltophilia* DSF (cis-Δ2-11-methyl-dodecenoic acid), 1 mM *Burkholderia cenocepacia* DSF (BDSF, cis-Δ2-dodecenoic acid), or 1 mM *Pseudomonas aeruginosa* DSF (PDSF, cis-Δ2-decenoic acid), and allowed to grow further for 3 h.

### Time-kinetic, quantitative proteome study

Methodology is provided in Supplementary File S1.

### Isolation of outer membrane vesicles

To obtain cell free culture supernatant from *S. maltophilia*, cells were pelleted by centrifugation at 6000 × g for 5 min, and the culture supernatant was filtered through a 0.2 μm V25 vacuum filter (Sarstedt, Numbrecht, GE). The OMVs were pelleted by ultracentrifugation at 100,000 × g for 1 h (Avanti J-30I, Beckman Coulter, Fullerton, CA). One milliliter of the filtered culture supernatant was spread onto an LB agar plate and incubated at 37°C for 24 h to confirm the absence of intact, living cells.

### OMV protein extraction and digestion

OMV proteins were extracted by dissolving the OMV pellet in 1 ml 8 M urea in 50 mM Tris-HCl pH 8. Proteins were precipitated with trichloroacetic acid (20%) and consequently the pellet was washed twice with ice-cold acetone, and finally dissolved in 2 M urea in 50 mM ammonium bicarbonate (pH 8). The protein concentration was assessed using the Coomassie Plus Bradford™ Assay kit (Thermo Scientific, San Jose, CA, US). Proteins were reduced with 10 mM dithiothreitol for 30 min at 60°C, alkylated with 20 mM iodoacetamide at ambient temperature for 30 min, and then digested with trypsin (1:50 w/w) overnight at 37°C.

### LCMS^E^ analysis of outer membrane vesicle content

Peptide mixtures (0.5 μg/μl in 100 mM ammonium formate, pH 10) were separated on a NanoAcquity UPLC® system (Waters Corporation, Milford, MA) in 2D mode. For the first dimension (high pH), solvent A1 and B1 were composed of 20 mM ammonium formate in water and ACN (pH 10), respectively. For the second dimension (low pH), solvent A2 and B2 were composed of 0.1% formic acid in water and 0.1% formic acid in ACN, respectively. The sample (1 μg) was loaded onto an Xbridge™ BEH130 C18 column (300 μm × 50 mm, 5 μm; Waters) at 3% solvent B1 at 2 μL/min. Peptides were eluted from the first dimension column in 5 fractions (11.1%, 14.5%, 17.4%, 20.8%, and 45.0% of solvent B1), and fractions were trapped on a Symmetry® C18 trapping column (180 μm × 20 mm, 5 μm; Waters). Each fraction was separated on a HSS T3 C18 analytical column (75 μm × 250 mm, 1.8 μm; Waters) at 40°C at 250 nL/min by increasing the acetonitrile concentration from 5 to 50% B2 over 60 min.

The outlet of the column was directly connected to a PicoTip Emitter (uncoated SilicaTip™ 10 ± 1 μm, New Objective, Woburn, MA, US) mounted on a Nanolockspray source of a SYNAPT™ G1 HDMS mass spectrometer (Waters). The time-of-flight (TOF) analyzer was externally calibrated with MS/MS fragments of human [glu^1^]-fibrinopeptide B (Glu-fib) from *m/z* 72 to 1285, and the data was corrected post-acquisition using the monoisotopic mass of the doubly charged precursor of Glu-fib (*m/z* 785.8426) (lock mass correction). Accurate mass data were collected in a data independent positive mode of acquisition (MS^E^) by alternating between low (5 V) and high (ramping from 15 to 35 V) energy scan functions (Geromanos et al., 2009). The selected *m/z* range was 125–2000 Da. The capillary voltage was set to 3.0 kV, the sampling cone voltage was 26 V and the extraction cone voltage on 2.65 V. The source temperature was set on 65°C.

The LCMS^E^ data were processed using the ProteinLynx Global SERVER™ v2.5 (PLGS, Waters Corporation) (Geromanos et al., 2009). In brief, lock mass-corrected spectra (0.250 Da window allowed) were automatically centroided, deisotoped and charge-state reduced to produce a single monoisotopic peak for each peptide and associated fragment ion. The correlation of a precursor and a potential fragment ion was achieved by means of time alignment. The following parameters were used for the data processing in PLGS: the chromatographic peak width, the TOF resolution and retention time window, which were determined automatically by the software, and the low energy, high energy, and intensity thresholds, which were set to 250, 100, and 1500 counts, respectively. A database containing 4380 protein entries from the closely related *Stenotrophomonas maltophilia* K279a (downloaded from the Uniprot website, April 2014), together with a decoy database consisting of the randomized entries of all the proteins, was interrogated by PLGS (Li et al., 2009). The precursor and fragment ion tolerance were determined automatically. The default protein identification criteria used included a maximal protein mass of 250,000 Da, a detection of minimal three fragment ions per peptide, minimal seven fragment ions per protein and minimal one peptide per protein. Carbamidomethyl-C (fixed) and methionine oxidation (variable) were selected as modifications. Maximally one missed cleavages and a false positive rate of 4% was allowed.

### Sample preparation for liquid chromatography-multiple reaction monitoring (LC-MRM) analysis

Cells, obtained from 10 ml of culture, were pelleted by centrifugation at 6000 × g for 5 min, and the culture supernatant was filtered through a syringe-driven 0.22 μm PES membrane filter unit (Merck Millipore, Darmstadt, GE). OMV proteins were isolated as described above, and finally dissolved in 50 μl 2 M urea in 50 mM ammonium bicarbonate. Protein solutions were spiked with 100 ng BSA (MS grade protein standard), reduced and alkylated, and digested with 0.5 μg trypsin. Digested samples were dried and dissolved in 50 μl 0.1% formic acid in water for LC-MRM analysis (5 μl injection). This procedure was followed for two biological replicates.

### LC-MRM analysis

The digested samples were first separated by RPLC on a U3000-RSLC system (Thermo). Briefly, the sample was loaded onto an Acclaim PepMap100 pre-concentration column (L × ID 2 cm × 100 μm, C18, 5 μm, 100 Å) at a flow rate of 5 μl/min, and flushed for 3 min with 0.1% HCOOH/2% ACN. The sample was then separated on a Thermo Acclaim PepMap100 analytical column (L × ID 15 cm × 75 μm, C18, 3 μm, 100 Å) at a flow rate of 300 nl/min, with mobile phases 0.1% HCOOH in water (solvent A) and 0.1% HCOOH in ACN (solvent B). Peptides were separated with a 30 min gradient, going from 2 to 40% solvent B, and eluting peptides were sprayed directly in a 4000 QTRAP mass spectrometer (AB Sciex, Framingham, MA) with a NanoSpray II ESI source (AB Sciex), using a PicoTip Emitter (uncoated SilicaTip™ 10 ± 1 μm). The ion spray voltage was set at 3.5 kV, curtain gas at 10 (arbitrary units), nebulizing gas at 5, and interface heater temperature at 60°C. We analyzed two technical replicates per biological samples.

Target peptides (two for each protein) were measured in multiple reaction monitoring (MRM) acquisition mode, with the Q1/Q3 resolution set at LOW and with a maximum total cycle time of 3 s. The double charged peptide was selected as precursor (Q1), and for each precursor three fragment ions were selected from the y-ions (Q3). The collision energy (CE) and declustering potential (DP) were calculated with the following equations:

$$\begin{array}{l} \text{CE}(\text{V})=(0.5\times \text{m}/\text{z})+5\\ \text{DP}(\text{V})=(0.0729\times \text{m}/\text{z})+31.117 \end{array}$$

The MRM data was imported in Skyline v2.5 (MacLean et al., 2010) and peak traces were subjected to a Savitzky-Golay Smoothing transformation. The total area under curve (AUC) of each target peptide was calculated, and normalized to the spiked BSA standard. A student's *t*-test was performed to evaluate the significance of differential protein abundancy levels between stimulated and unstimulated cultures.

### OMV quantification

OMVs from 25 ml cultures were harvested as described above, and the pellet was dissolved in 100 μl phosphate buffered saline (PBS). The OMV concentration and size was determined by light scattering based single particle tracking using a NanoSight LM10-HS instrument (NanoSight, Amesbury, UK) equipped with a 405 nm laser. Prior to analysis, the purified OMVs were diluted in PBS-buffer (Invitrogen). Movies of 60 s were recorded and analyzed with the NTA Analytical Software version 2.3. Each individual sample was diluted and measured three times. Calculations were performed according to Van der Pol et al. (2010).

### Transmission electron microscopy

The *S. maltophilia* strain 44/98 was cultured overnight, as described above. At the mid exponential growth phase, cultures were diluted in LB medium supplemented with 25 μg/mL imipenem, and allowed to grow further for 2 h. A control culture was treated similarly except for the addition of imipenem. A 4 μL drop of culture was placed on a Formvar/carbon-coated cupper grid, made hydrophilic by glow discharging for 30 s. The grid was then washed by placing it sequentially onto five drops of milliQ water. After these washing steps, the grid was placed on two drops of 2% uranyl acetate and incubated for 30–40 s. The grid was blotted with filter paper between each washing step. The created specimens were examined using a JEM 1010 transmission electron microscope (JEOL, Tokyo, Japan) operating at 60 kV using Image Plate Technology from Ditabis (Pforzheim, Germany).

## Results

### Imipenem stimulates the expression of two Ax21 homologs

**Figure 1:**
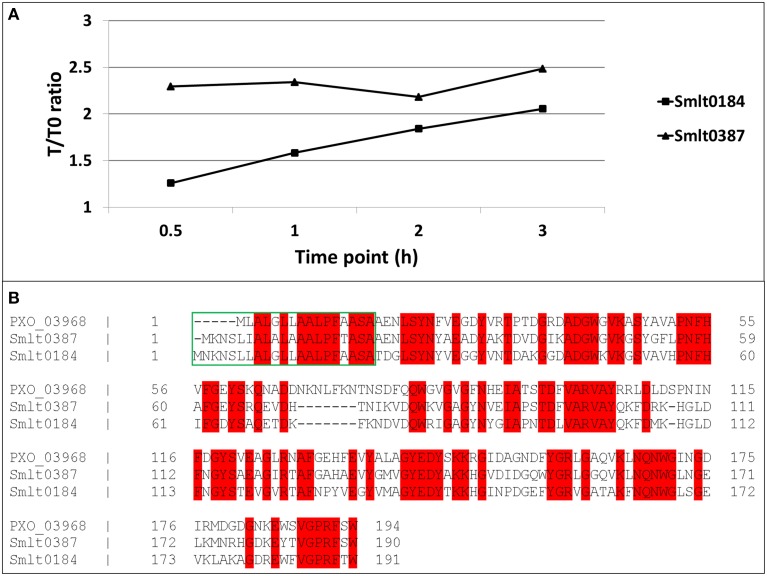
**(A)** Time-dependent increase in abundancy of the two *S. maltophilia* Ax21 homologs (Smlt0387 and Smlt0184). For each time point, the abundance ratio with reference to time point zero is plotted. **(B)** Alignment of Smlt0387 and Smlt0184 with the *Xanthomonas* Ax21 protein (PXO_03968). Red: identical amino acid residues. Green box: signal sequence.

A preliminary time-kinetic, quantitative proteome study on the imipenem response of *S. maltophilia* was performed to address the proteome dynamics after antibiotic treatment. Among the few proteins of which we found a gradual increase in abundancy over time after exposure to imipenem, we found mainly the two β-lactamases, outer membrane proteins and proteins involved in motility (flagellins) (Supplementary File S2). Two of these proteins showed strong homology to *X. oryzae* Ax21 (PXO_03968), i.e., Smlt0387 and Smlt0184 (Figure 1A). Former studies on Ax21 in *S. maltophilia* report only a single Ax21 homolog (Smlt0387) (McCarthy et al., 2011; Ferrer-Navarro et al., 2013). We found now thus two homologs with, respectively, 60% identity for Smlt0387 (*e*-value 7.0e-80) and 56% identity for Smlt0184 (*e*-value 4.0e-73) with the *X. oryzae* protein. Alignment of Smlt0387 and Smlt0184 learned that the two proteins display 63% identity. The alignment of the two *S. maltophilia* Ax21 sequences with the *X. oryzae* Ax21 sequence is depicted in Figure 1B. Both proteins contain a typical signal sequence (Figure 1B, green box) implicating that, like the *X. oryzae* Ax21 protein, they are processed by the Sec secretion system (Bahar et al., 2014). Analyzing the protein sequences of the *S. maltophilia* Ax21 homologs Smlt0387 and Smlt0184 with Pfam (Finn et al., 2014) showed that they belong to the outer membrane protein β-barrel domain family, with *e*-values 1.5 e-09 and 5.7 e-11, respectively. This is consistent with the structural model, representing a porin-like structure, proposed recently (Park et al., 2014).

### Imipenem-treated *Stenotrophomonas maltophilia* cells secrete more OMVs

**Figure 2:**
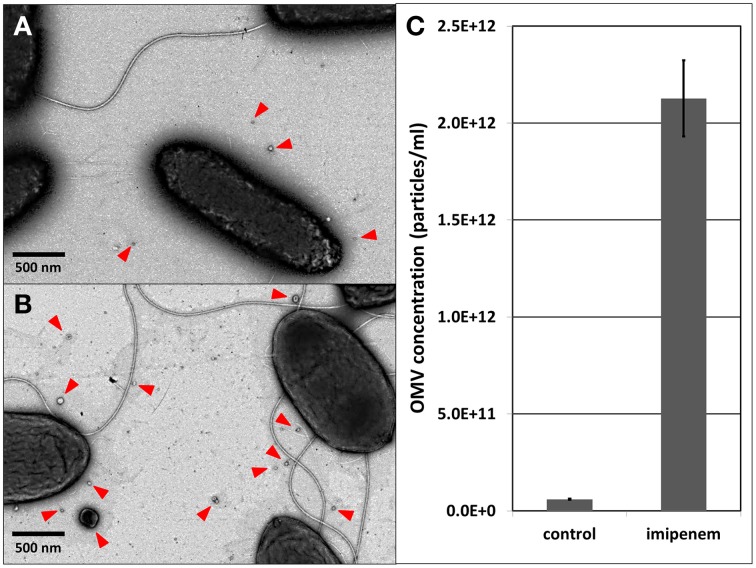
**OMV visualization with TEM: (A) control, (B) after imipenem treatment**. OMV concentration determination with light scattering based single particle tracking after imipenem treatment **(C)**. Error bars plot the SD.

A recent study demonstrated that the *X. oryzae* Ax21 protein is secreted in association with OMVs (Bahar et al., 2014). Therefore, we verified the production of OMVs by *S. maltophilia* upon imipenem stress. As a matter of fact, it was shown in *Acinetobacter baumanii* (ATCC19606^T^) that exposure to subinhibitory concentrations of ceftazidime (cephalosporine, also a β-lactam) caused ruffling along the whole outer membrane resulting in the formation of more OMVs (Koning et al., 2013). Using TEM, we indeed observed a similar increase in OMV secretion in *S. maltophilia*, when exposed to imipenem (Figures 2A,B). When we applied light scattering based single particle tracking to quantify the isolated OMVs from an equal volume of stimulated and unstimulated cultures (25 ml culture, 3 h imipenem stimulation), the stimulated cultures contained considerable more OMVs than the unstimulated cultures (Figure 2C; Supplementary File S3).

### Proteomic analysis of *S. maltophilia* OMVs

To confirm the presence of both Ax21 proteins in OMVs, a profile of the *S. maltophilia* OMV proteome was assessed with LCMS^E^. Cells were grown in the presence of the broad-spectrum β-lactam antibiotic imipenem (3 h). After collection of the OMVs and protein extraction, one microgram of digested protein was separated with 2D-UPLC (high pH–low pH RPLC) on a NanoAcquity UPLC® system, and eluting peptides were analyzed online with a SYNAPT™ HDMS Q-TOF mass spectrometer. The acquisition of MS/MS spectra in a data-independent acquisition mode (MS^E^) resulted in the identification of 234 proteins (identified in at least two of the three technical replicate LCMS^E^ runs) (Supplementary File S4). Indeed, the Ax21 proteins Smlt0387 and Smlt0184 were identified in all three technical replicate runs with, on average, a sequence coverage of 82.1% and 70.0%, respectively. These findings confirm the secretion of Ax21 as an outer membrane protein associated with outer membrane vesicles in *S. maltophilia*.

The proteins identified in the OMVs were further annotated with Blast2GO v.2.7.1 and PSORTb v.3.0.2 (Supplementary File S4). Several proteins were identified that are involved in the assembly of β-barrel proteins: SecA and SecB for protein translocation to the periplasm, the SurA chaperone which prevents mis-folding in the periplasm, and BamA, BamB, BamD and BamE, responsible for β-barrel protein assembly in the outer membrane (Selkrig et al., 2013). Apart from the Ax21 homologs, several other outer membrane proteins were identified, for example TonB-dependent receptor proteins, Omp family proteins, autotransporters, lipoproteins, and the SmeX efflux protein (part of the Resistance-Nodulation-Cell Division (RND) SmeVWX efflux pump). During OMV formation, periplasmic proteins can be encapsulated, as well as inner membrane (associated) proteins. This is illustrated by the identification of proteins involved in peptidoglycan turnover (penicillin-binding proteins (PBPs), carboxypeptidases, transglycosylases). Furthermore, fimbrial adhesins, and flagellins were identified, involved in adhesion and motility, respectively. Interestingly, the OMVs also contain the L1-metallo- and L2-serine-β-lactamases (Smlt2667 and Smlt3722) when exposed to imipenem. Both β-lactamases are translocated to the periplasm via different systems: the L1-β-lactamase uses the Sec export system, the L2-β-lactamase uses the Tat export system (Pradel et al., 2009; Brooke, 2012). Finally, the OMV proteome profile also includes cytoplasmic proteins, mostly highly abundant proteins (e.g., elongation factors) and ribosomal proteins.

### Influence of diffusible signaling factors on the secretion of OMVs

**Figure 3:**
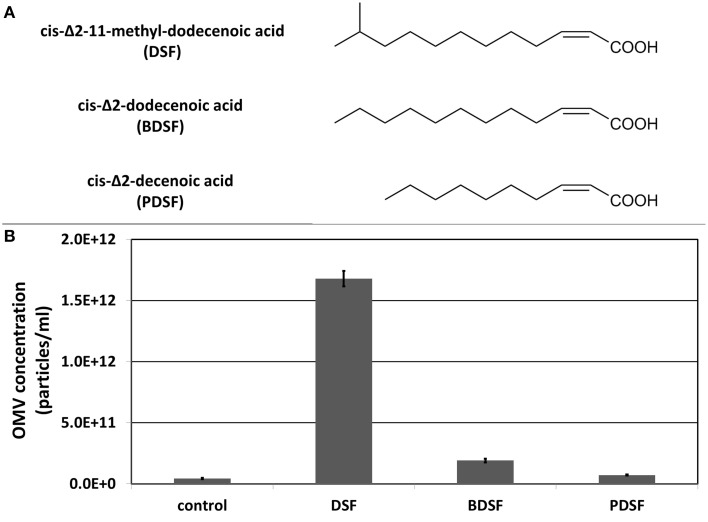
**(A)** Structure of DSF, BDSF, and PDSF. **(B)** Effect of DSF and its structural homologs on OMV secretion determined with light scattering based single particle tracking. Error bars plot the SD.

Next to the β-lactam antibiotic imipenem, also DSF quorum sensing molecules were tested for their ability to stimulate OMV secretion (Figure 3A). A study on the plant pathogen *Xylella fastidiosa* showed a link between OMV secretion and the DSF quorum sensing system (Ionescu et al., 2014). The authors postulated that OMVs are affecting plant colonization by blocking surfaces, leading to a deeper spread of *X. fastidiosa* into the plant host, which increases virulence. The *X. fastidiosa* DSF system suppresses the release of OMVs (and virulence), causing cells to grow more locally, attached to unblocked surfaces. However, in *S. maltophilia*, growing cells in presence of its own DSF cis-Δ2-11-methyl-dodecenoic acid resulted in a remarkable increase in OMV secretion (Figure 3B), comparable to the amount secreted in the presence of imipenem. The DSF cis-Δ2-dodecenoic acid produced by *B. cenocepacia* (BDSF) also led to a slight increase in OMV secretion in *S. maltophilia*, while the DSF cis-Δ2-decenoic acid produced by *P. aeruginosa* (PDSF) did not. This is in accordance with the known responsiveness of *S. maltophilia* to these DSF molecules. *S. maltophilia* can perceive the DSFs produced by itself and *B. cenocepacia*, but not the one produced by *P. aeruginosa* (Ryan and Dow, 2010). The endogenous methyl-branched DSF was shown to be far more active than the unbranched BDSF and PDSF.

### Relative quantification of OMV-associated proteins with multiple reaction monitoring (MRM)

The differential OMV-associated secretion of the Ax21 homologs was validated with a label-free, targeted LC-MRM approach. Two proteotypic peptides for each Ax21 protein were quantitatively analyzed with MRM: VGAGYNVEIAPSTDFVAR and LNQNWGLNGELK for Smlt0387, and IGAGYNYGIAPNTDLVAR and FNQNWGLSGEVK for Smlt0184. Three MS/MS transitions for each peptide were chosen (Supplementary File S5). The peptides are unique within the *S. maltophilia* K279a proteome, and a UniPept search (Mesuere et al., 2012) also revealed uniqueness for *S. maltophilia* species. These peptides also show good MRM compatibility in terms of length, hydrophobicity, and ionization properties. In addition, the dominant L1 β-lactamase (Smlt2667) (target peptides GVAPQDLR and IAYADSLSAPGYQLK) and the spiked-in BSA standard (target peptides AEFVEVTK and QTALVELLK) were monitored. The MRM analysis was performed on OMV protein extracts from equal culture volumes grown in presence of imipenem, DSF, BDSF, and PDSF (3h stimulation).

**Figure 4:**
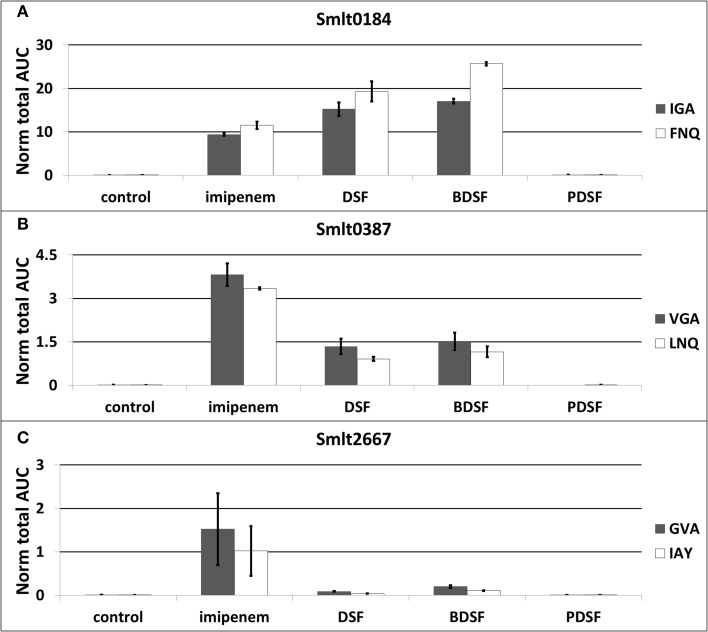
**Relative abundancy of OMV-associated Ax21 homologs (A,B) and L1 β-lactamase (C) in response to imipenem and diffusible signaling factors, determined by targeted proteomics (LC-MRM)**. The plot display the average normalized area-under-the-curve for each peptide used as marker for the different proteins. The error bars plot the SD of all replicate analyses. Target peptides: VGA: VGAGYNVEIAPSTDFVAR, LNQ: LNQNWGLNGELK, IGA: IGAGYNYGIAPNTDLVAR, FNQ: FNQNWGLSGEVK, GVA: GVAPQDLR, IAY: IAYADSLSAPGYQLK.

These experiments confirmed a huge increase in OMV-associated secretion of both Ax21 proteins, when *S. maltophilia* cultures were stimulated with the β-lactam antibiotic imipenem, and with the DSF and BDSF quorum sensing molecules (Figures 4A,B; Supplementary File S5). When comparing the two Ax21 homologs, the previously overlooked homolog Smlt0184 is actually much more prevalent than Smlt0387, both in the imipenem-stimulated culture, as well as in the DSF- and BDSF-stimulated cultures. Also remarkable is that the highest amount of Smlt0184 was measured in the BDSF-induced OMVs (Figure 4A), while the OMV production elicited by BDSF is in fact much lower than for imipenem and DSF (Figure 4B). Smlt0387 secretion seems to be more pronounced after imipenem exposure, than it is for DSF or BDSF (Figure 4B). *S. maltophilia* is again unresponsive to the *P. aeruginosa* PDSF in terms of Ax21 secretion, as it was for OMV production (Figure 4B).

The large increase in L1 β-lactamase expression in response to imipenem is also represented here in the secreted OMVs (Figure 4C; Supplementary File S5), as previously observed at the cellular level (Van Oudenhove et al., 2012). The fast production of β-lactamases, especially the L1 metallo-β-lactamase, represents the important early line of defense of *S. maltophilia* against the imipenem challenge. Since imipenem leads to substantial L1 levels in the cell, and the periplasm, it is not entirely surprising that this protein is also prevalent in the secreted OMVs. Nevertheless, whether its presence in the OMVs is accidental or predestined, its biological relevance is obvious. Finally, only a slight increase in OMV-associated L1 secretion was observed when stimulated with DSF, and even more with BDSF (Figure 4C).

## Discussion

The genome sequence of the pathogenic *Stenotrophomonas maltophilia* K279a strain revealed an organism that is well adjusted for living in an environment with antibiotics (Crossman et al., 2008). We here add the capacity of *S. maltophilia* to secrete OMVs packed with β-lactamases as an additional property to adapt to antibiotic stress.

In this work, we quantified the amount of OMV secretion as a response to the β-lactam antibiotic imipenem. As expected, imipenem led to a significant increase in OMV secretion, probably owing to the disturbed cell wall structure or alteration in peptidoglycan dynamics (Haurat et al., 2014). Proteomic analysis on the isolated OMVs revealed the OMV-mediated secretion of the chromosomal encoded β-lactamases in *S. maltophilia*. This is not the case when OMVs are induced by DSF. Whether the presence of β-lactamases in the OMVs is merely due to their high abundance in the periplasm upon β-lactam stress or whether they are deliberately delivered in the OMVs is not clear. Anyway, by exporting the β-lactamases in the environment, *S. maltophilia* not only provides resistance against the imipenem at the cell level, but could also protect other cells from the same species, or from other species. Additionally, packed in OMVs, the β-lactamases are protected against extracellular degradative enzymes and are able to travel long distances (Bonnington and Kuehn, 2013).

In *Xylella fastidiosa*, the production of OMVs is suppressed by the DSF quorum sensing system (Ionescu et al., 2014). Therefore, the DSF quorum sensing molecules cis-Δ2-11-methyl-dodecenoic acid (DSF), cis-Δ2-dodecenoic acid (BDSF) and cis-Δ2-decenoic acid (PDSF), produced by *S. maltophilia*, *B. cenocepacia* and *P. aeruginosa*, respectively, were also tested for their effect on OMV production in *S. maltophilia*. In contrast to *X. fastidiosa*, DSF led to a comparable increase in the amount of OMV secretion as with imipenem. BDSF led to a slight increase in OMV secretion, and PDSF did not have any effect. These fatty acid analogs do not have any known perturbation effects on the cell wall, as opposed to imipenem, and the OMV response is in agreement with their signaling activity in *S. maltophilia*. These results therefore suggest a quorum sensing controlled OMV biogenesis.

Finally, the OMV proteome analysis revealed the production of two Ax21 proteins in *S. maltophilia*. The Ax21 protein was shown to be involved in biofilm formation and virulence, and is highly conserved in all *Xanthomonas* species, and in *S. maltophilia*. Recently, it was shown that the Xanthomonas Ax21 is an outer membrane protein, secreted in OMVs (Bahar et al., 2014), and regulation of expression is dependent on the DSF system (Qian et al., 2013). With a targeted and label-free MRM method, we quantified the OMV-mediated secretion of the Ax21 homologs as a response to imipenem, DSF, BDSF, and PDSF. All conditions led to substantial amounts of OMV-associated Ax21 secretion. The results indicate a deliberate and regulated secretion of Ax21, rather than a coincidental presence due to an increased OMV biogenesis. The production of OMVs and packing with large quantities of Ax21 protein, point to an important role of it in antibiotic resistance and biofilm formation. However, the exact role of Ax21 in *S. maltophilia* remains unclear, and should be further investigated, more specific by confirming its porin transporter function and determination of its cargo.

## Author contributions

SD performed the 2D-LCMS^E^ OMV profiling experiment, as well as the LC-MRM analysis. LV performed the time-kinetic, quantitative proteome study. GV provided technical support for all measurements with the NanoAcquity UPLC® system and the SYNAPT™ G1 HDMS mass spectrometer. SS carried out the OMV quantification experiments, with help of JV, and supervision of KR. WVP did the TEM imaging of OMVs, under supervision of RD. SD drafted the manuscript. BD supervised the research and edited the manuscript.

### Conflict of interest statement

The authors declare that the research was conducted in the absence of any commercial or financial relationships that could be construed as a potential conflict of interest.

## Supplementary material

Supplementary File S1 **Material and methods for the time-kinetic, quantitative proteomics study of the response of *Stenotrophomonas maltophilia* to imipenem**.

Supplementary File S2 **Results from the time-kinetic, quantitative proteomics study of the response of *Stenotrophomonas maltophilia***. Samples were taken at 0, 0.5, 1, 2, and 3h after treatment with imipenem. Table 1 contains the results for proteins for which a significant change in abundance at any of the time points was determined. Table 2 contains raw LCMS^E^ results.

Supplementary File S3 **OMV concentration determined by light scattering based single particle tracking**.

Supplementary File S4 **Results of the proteomic analysis of OMVs (Table 1: summary, Tables 2–4: data for the three replicate analyses)**.

Supplementary File S5 **List of transition used for LC-MRM analysis (Table 1) and results of the LC-MRM analysis of the two AX21 proteins (Tables 2, 3) and beta lactamase L1 (Table 4)**. Table 5 contains the statistical analysis of the data for all proteins.
