# Supplementary material for: The effect of imipenem and diffusible signaling factors on the secretion of outer membrane vesicles and associated Ax21 proteins in Stenotrophomonas maltophilia
Source: Front Microbiol. 2015 Apr 14;6:298. doi: 10.3389/fmicb.2015.00298 (PMC4396451; doi:10.3389/fmicb.2015.00298)
Supplement: Supplementary File S1 — Material and methods for the time-kinetic, quantitative proteomics study of the response of Stenotrophomonas maltophilia to imipenem. [file DataSheet1.DOCX]

**S1. Material and methods for the time-kinetic, quantitative proteomics study of the response of *Stenotrophomonas maltophilia* to imipenem**

**Materials**

Urea was obtained from GE Healthcare (Diegem, Belgium). ‘Complete mini’ EDTA-free protease inhibitor mix was purchased from Roche Diagnostics (Vilvoorde, Belgium). Rapigest detergent and the rabbit glycogen phosphorylase B standard peptide mixture were from Waters Corporation (Milford, MA, US). Sequencing grade modified trypsin was obtained from Promega (Madison, WI, US), while mass spectrometry grade lysyl endopeptidase was from Wako Chemicals (GmbH, Neuss, Germany). ULC-MS grade water, acetonitrile (ACN) and formic acid was procured from Biosolve (Valkenswaard, The Netherlands). Imipenem was kindly donated by Prof. M. Galleni (CIP, University of Liège, Belgium). Other chemicals and reagents were purchased from Sigma-Aldrich (St. Louis, MO, US).

**Bacterial cell culture**

The imipenem-resistant *S. maltophilia* strain 44/98 (BCCM/LMG bacteria collection, LMG 26824) was isolated at the Clinical Microbiology Unit of the Varese University hospital in Italy. Cells were grown aerobically overnight as two separate cultures until the stationary phase. The cell suspensions were then separately diluted 10-fold in 300 mL of fresh Luria Broth (LB) medium, and allowed to grow further until they reached the mid-exponential growth phase (OD_600nm_ = 0.65-0.75). From these two culture flasks, samples were harvested by centrifugation at 2,500 x g for 5 min (Time 0). The remaining cultures were stimulated with 25 μg/mL imipenem. Samples were also harvested 30 minutes, 1 h, 2 h and finally 3 h after the imipenem challenge (Time 0.5, 1, 2 and 3 respectively, each in duplicate).

**Sample preparation for LCMS^E^**

The pellets were resuspended in a solution containing 6 M urea, 2 M thiourea, 0.2% Rapigest, and an EDTA-free protease inhibitor mixture in 50 mM ammonium bicarbonate. The cells were disrupted with acid-washed glass beads (diameter of 1-1.25 mm) for 180 s in a Precellys 24 instrument (Bertin Technologies, Orléans, France). The protein solutions were cleaned-up and concentrated by acetone precipitation, followed by resuspension and rinsing on a 10 kDa cut-off spin column (Ultracel Amicon, Millipore, MA, US) with 0.2% Rapigest, 1 mM DTT and 50 mM ammonium bicarbonate as the buffer. The protein concentration was assessed using the Coomassie Plus Bradford^TM^ Assay kit (Thermo Scientific, San Jose, CA, US). Ten μg of each protein extract was heated at 80 °C for 15 min, reduced with 2.5 mM DTT for 45 min at 60 °C and subsequently alkylated with 7.5 mM iodoacetamide at ambient temperature for 30 min. The proteins were first digested with 1:50 (w/w) lysyl endopeptidase for 3 h at ambient temperature, followed by trypsin digestion (1:50 w/w) overnight at 37 °C. The Rapigest detergent was hydrolyzed by the addition of formic acid to the solution, and removed by centrifugation. Each sample was diluted with a glycogen phosphorylase B standard peptide mixture and 0.1% formic acid in water to give a final protein concentration of 0.5 μg/μl per sample and 50 fmol/μl phosphorylase B.

**LCMS^E^ analysis**

The peptide mixtures were separated on a NanoAcquity UPLC^®^ system (Waters Corporation) using a Symmetry^®^ C18 trapping column (180 μm x 20 mm, 5 μm) and a BEH^TM^ C18 analytical column (75 μm x 250 mm, 1.7 μm) at 40 °C. Solvent A and B were composed of 0.1% formic acid in water and 0.1% formic acid in ACN, respectively. Each sample (0.5 μg of total protein and 50 fmol of phosphorylase B) was loaded onto the trapping column with 0.1% solvent B for 1 min at 15 μL/min and eluted at 250 nL/min by increasing the organic solvent concentration from 3-40% B over 90 min. Analyses were performed in quadruplicate. The eluting peptides were directly ionized and analyzed with a SYNAPT^TM^ HDMS using a PicoTip Emitter from New Objective (uncoated silicaTip^TM^ 10 +/- 1 μm, Woburn, MA, US). The time-of-flight (TOF) analyzer was externally calibrated with MS/MS fragments of human [glu^1^]-fibrinopeptide B (GFP) from *m/z* 72 to 1285, and the data was corrected post-acquisition using the monoisotopic mass of the doubly charged precursor of GFP (*m/z* 785.8426), which was measured with a collision energy of 6.0 V and sampling cone voltage of 45 V (lockmass correction). The GFP was delivered at 500 fmol/μL to the mass analyzer by a NanoLockSpray interface using the auxiliary pump of the NanoAcquity system at a flow rate of 300 nL/min. The reference sprayer was sampled every 30 s. Accurate mass data were collected in a data independent positive mode of acquisition (MS^E^) from 15 to 120 minutes by alternating between low (5 V) and high (ramping from 15 to 35 V) energy scan functions. The spectral acquisition scan rate was 0.48 s with a 0.1 s inter-scan delay. The selected *m/z* range was 125 to 2000 Da. The capillary voltage was set to 3.0 kV, the sampling cone voltage was 26 V and the extraction cone voltage on 2.65 V. The source temperature was set on 65 °C.

**Identification of proteins**

The LCMS^E^ data were processed using the ProteinLynx Global SERVER^TM^ v2.5 (PLGS, Waters Corporation). In brief, lockmass-corrected spectra (0.250 Da window allowed) were automatically centroided, deisotoped and charge-state reduced to produce a single monoisotopic peak for each peptide and associated fragment ion. The correlation of a precursor and a potential fragment ion was achieved by means of time alignment, in the first instance. The following parameters were used for the data processing in PLGS: the chromatographic peak width, the TOF resolution and retention time window, which were determined automatically by the software, and the low energy, high energy, and intensity thresholds, which were set to 250, 100 and 1500 counts respectively. A database containing 4369 protein entries from the closely related *Stenotrophomonas maltophilia* K279a (Uniprot website, June 2011), phosphorylase B (spiked into each sample) and potential contaminating proteins, as well as the randomized entries of all the proteins was interrogated by PLGS. The precursor and fragment ion tolerance were determined automatically. The default protein identification criteria used included a maximal protein mass of 500,000 Da, a detection of minimal 3 fragment ions per peptide, minimal 7 fragment ions per protein and minimal 1 peptide per protein. Fixed modification of carbamidomethyl-C and the variable modifications included acetylation (N-terminus), deamidation (N/Q) and oxidation of the methionines were selected. Maximally two missed cleavages and a false positive rate of 4% was allowed. The quality of the LCMS^E^ runs was examined with MassLynx v 4.1 (Waters Corporation), IBM^®^ SPSS Statistics v 19, and Excel.

**Label-free quantitative analysis with Expression^E^ from PLGS**

A relative quantification analysis of the protein abundances at the different time points, before and after the antibiotic challenge, was performed using the Expression^E^ software integrated in PLGS v.2.5. The algorithm uses the weighted sum of the peak intensities of all peptides for the relative quantification of the proteins. The contribution of a peptide intensity in the protein quantification is based on the uniformity of the intensities of this peptide in the technical replicates, as well as the peptide identification score. Protein ratios were normalized to the intensity of the dominant background of proteins showing no change in abundance between the different time collections. The proteins had to be identified in at least 2 technical replicates, with a score of 150 and a probability of 95%, as well as a confidence limit of maximally 2.5. The significance level of regulation was set at 30% fold change, corresponding to an average relative fold change of -0.3 and 0.3 on a natural log scale. This is 2-3 times higher than the estimated error on the intensity measurement. The results were exported to Excel and IBM^®^ SPSS Statistics v 19 for further analysis.
